# Association between two distinct executive tasks in schizophrenia: a functional transcranial Doppler sonography study

**DOI:** 10.1186/1471-244X-6-25

**Published:** 2006-05-24

**Authors:** Deborah Feldmann, Daniel Schuepbach, Bettina von Rickenbach, Anastasia Theodoridou, Daniel Hell

**Affiliations:** 1 Psychiatric University Hospital Zürich, Lenggstrasse 31, 8032 Zürich, Switzerland

## Abstract

**Background:**

Schizophrenia is a severe mental disorder involving impairments in executive functioning, which are important cognitive processes that can be assessed by planning tasks such as the Stockings of Cambridge (SOC), and tasks of rule learning/abstraction such as the Wisconsin Card Sorting Test (WCST). We undertook this study to investigate the association between performance during separate phases of SOC and WCST, including mean cerebral blood flow velocity (MFV) measurements in chronic schizophrenia.

**Methods:**

Functional transcranial Doppler sonography (fTCD) was used to assess bilateral MFV changes in the middle (MCA) and anterior (ACA) cerebral arteries. Twenty-two patients with chronic schizophrenia and 20 healthy subjects with similar sociodemographic characteristics performed SOC and WCST during fTCD measurements of the MCA and the ACA. The SOC was varied in terms of easy and difficult problems, and also in terms of separate phases, namely mental planning and movement execution. The WCST performance was assessed separately for maintaining set and set shifting. This allowed us to examine the impact of problem difficulty and the impact of separate phases of a planning task on distinct intervals of WCST. Simultaneous registration of MFV was carried out to investigate the linkage of brain perfusion during the tasks.

**Results:**

In patients, slowing of movement execution during easy problems (SOC) was associated with slowing during maintaining set (WCST) (P < 0.01). In healthy subjects, faster planning and movement execution during predominantly difficult problems were associated with increased performance of WCST during set shifting (P < 0.01). In the MCA, patients showed a significant and positive correlation of MFV between movement execution and WCST (P < 0.01).

**Conclusion:**

The results of this study demonstrate performance and brain perfusion abnormalities in the association pattern of two different tasks of executive functioning in schizophrenia, and they support the notion that executive functions have a pathological functional correlate predominantly in the lateral hemispheres of the brain. This study also underpins the scientific potential of fTCD in assessing brain perfusion in patients with schizophrenia.

## Background

In recent years, several studies have described deficits in abstraction, mental flexibility and planning, i.e. executive functions, in patients with schizophrenia [1,2]. The underlying functional substrate has been examined by investigating changes in cerebral perfusion during performance of paradigms such as the Wisconsin Card Sorting test (WCST) [3] and the Tower of London (TOL) [4]. Weinberger et al. [5] found evidence of lower cerebral blood flow (CBF) in prefrontal regions during WCST, and Andreasen et al. [6] observed lower frontal activation during the TOL. Most of the aforementioned studies have used only one paradigm to examine executive function in schizophrenia, limiting inferences on pathophysiological substrate to the psychometric properties of the applied test. Yet, there is consensus that the term "executive function" is multifaceted, referring to complex cognitive functions that connect and control the flow of information between cognitive subsystems, monitoring perceptual inputs and coordinating goal-oriented activity [4,7]. Hence, it would be interesting to investigate the linkage between a complex test of executive function such as the WCST, and a somewhat more specific cognitive task in that domain, the Stockings of Cambridge (SOC), a related version of the TOL. One of the appealing features of SOC consists in the fact that distinct phases during a planning process, such as mental planning and execution of a plan, can be assessed. Morris et al. [1] found no significant difference in planning time between patients with schizophrenia and healthy controls, but a deficit in accuracy and execution time in patients – results similar to those in patients with frontal lobe lesions [8]. Therefore, these authors suggested a frontal lobe involvement during planning within a complex cerebral dysfunction in this disorder. To the best of our knowledge, there are no studies to date that have investigated the association of performance/brain perfusion between separate phases of a planning task and an abstraction/rule generation task such as the WCST in schizophrenia. The examination of such relationships is interesting because the WCST is a complex multidimensional task that has frequently been applied in schizophrenia [5]. Particularly, two distinct and alternating phases of WCST have been described in terms of performance and in terms of neurophysiological correlates [9]: the search for a new principle (set shifting) and maintaining a rule (maintaining set). Reaction time (RT) increases during set shifting as compared to maintaining set, reflecting more complex cognitive processes such as the need to change the rule and the inhibition of other rules [9,10]. Based on findings during difficult planning problems of SOC in healthy subjects, where a disproportionate increase in execution time and hence replanning or online planning was observed [11], one may hypothesize that set shifting during WCST implements features of a replanning strategy, such as task-set activation and inhibition. Morice and Delahunty [2], examining WCST, TOL and other tasks of working memory (WM) in schizophrenia, suggested a failure to acquire the ability to process complex information.

**Figure 1:**
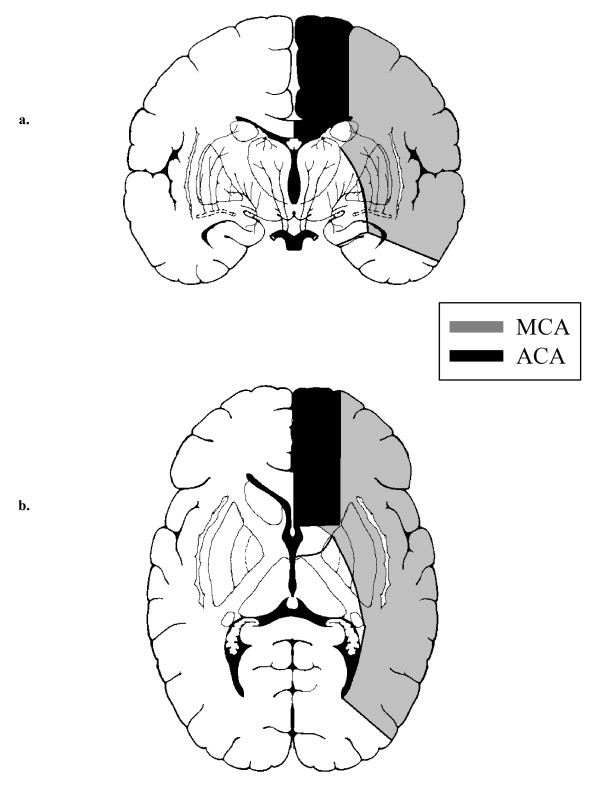
**Arterial territories of the middle (MCA) and anterior (ACA) cerebral arteries. a**. Coronal view, **b**. Axial view.

Functional transcranial Doppler sonography (fTCD) has been shown to detect specific alterations in MFV during the WCST [10,12,13], the Tower of Hanoi (TOH) [12] and the SOC [11]. In a study in patients with schizophrenia, preliminary results suggested reduced MFV in the area of the MCA during performance of the TOH [14]. Cognitive activation studies showed very good agreement between fTCD and functional magnetic resonance imaging (fMRI) [15]. Additional information on fTCD during cognitive challenge has been provided by two extensive reviews [16,17]. Particular advantages of fTCD are good temporal resolution, low restriction and low costs. Concerning research on cognitive paradigms, a major advantage is the fact that cognitive processes do not have to be synchronized to the technical specifications of the measurement apparatus. It is of relevance to note that the region of the MCA comprises lateral parts of the frontal, parietal and temporal lobes, whereas that of the ACA involves the medial part of the frontal and parietal lobes, including the frontal pole and also the cingulate gyrus [18] (Fig. 1).

This study addressed the following issues: a) What specific association patterns exist between the performance parameters of SOC and WCST? b) Are there different association patterns for schizophrenic patients and healthy subjects? c) What kind of associations between MFV in the relevant paradigms can be found? d) Are there distinct association patterns for patients and healthy subjects?

## Methods

### Subjects

Twenty-two right-handed patients with chronic schizophrenia according to DSM-IV criteria (chart review) were included in this study. Subjects' characteristics are presented in Table 1. All were inpatients and under stable antipsychotic medication (amisulpride, clozapine, flupentixol, olanzapine, quetiapine, promazine, risperidone, zuclopenthixol). One patient received an antidepressant (paroxetine). The concomitant medication consisted of biperiden hydrochloride. The following exclusion criteria applied to these patients: 1) affective or organic brain disorders, 2) substance abuse for the last 3 months prior to the examination or a lifetime diagnosis of substance dependence, including a positive urine test for psychotropic substances, 3) mental retardation, 4) migraine and other headaches. Within 24 hours of fTCD measurements, patients were clinically assessed by means of the Brief Psychiatric Rating Scale (BPRS) [19] and the Clinical Global Impression Scale (CGI) [20]. Secondary effects of antipsychotics on the extrapyramidal system were examined with the Extrapyramidal Symptom Scale (EPS) [21] and the Barnes Akathisia Scale (BAS) [22]. Twenty right-handed healthy volunteers were included with sociodemographic features similar to the patients, and with known SOC performance and brain perfusion characteristics [11]. Healthy subjects showed somewhat higher vocabulary knowledge (Table 1). All subjects were free of medication, general medical, neurological and psychiatric illness. They were not allowed to consume caffeine or nicotine 2 hours prior to the fTCD examination, and they were not familiar with the contents of the study. Further, all participants denied any recent traumatic burden. All subjects gave their written informed consent, and the ethic committee of the Kanton of Zürich/specialised subcommittee (Psychiatry, Neurology and Neurosurgery) approved the study.

**Table 1:** Sociodemographics of patients with schizophrenia and healthy subjects

	Patients with Schizophrenia (n = 22)	Healthy Individuals (n = 20)	χ^2 ^or *t*
*Sociodemographic variables*			
Age (yrs)	34.9 (7.5)	31.0 (7.8)	1.70
Gender (male/female)	15/7	9/11	2.30
Education (yrs)	14.0 (3.5)	16.6 (2.6)	2.67*
Parental education	11.7 (3.4)	12.8 (3.0)	1.16
Premorbid IQ^1^	106.6 (14.6)	116.7 (17.5)	2.02*
			
*Patient's history*			
Age at onset of illness (yrs)	24.8 (6.5)	N/A	
Duration of illness (yrs)	10.4 (7.6)	N/A	
			
*Symptom severity*			
BPRS total score (average)	42.3 (11.9)	N/A	
CGI	4.4 (0.9)	N/A	
			
*Extrapyramidal side effects*			
EPS total score	6.0 (4.2)	N/A	
BAS score (global)	1.0 (1.1)	N/A	
			
*Average medication dose*			
Antipsychotic (cpe^2^)	524.8 (239.3)	N/A	

Values are mean (SD)

*P < 0.05

^1^MWT-A: Mehrfachwahl-Wortschatz-Test [48], equivalent of the National Adult Reading Test (NART) [49]

^2^average chlorpromazine-equivalent units

Abbreviations: BAS: Barnes Akathisia Scale; BPRS: Brief Psychiatric Rating Scale; EPS: Extrapyramidal Rating Scale; N/A: not applicable; yrs: years

### Technical procedures

This procedure has been described elsewhere [11-14]. Briefly, fTCD measurements were carried out with a Multi-Dop T TCD instrument (DWL Elektronische Systeme GmbH, Sipplingen, Germany), and two 2 MHz transducers were attached and fixed with a headband. The MCAs were insonated at a depth of 48–55 mm and both ACAs at a depth of 60–70 mm through the temporal bone window [23]. Peak MFV was assessed in all examined vessels. The order of MCA and ACA insonation was random. The SOC and WCST were conducted with a personal computer (Compaq Computer Inc., US), which was connected to a touch-screen monitor (this feature only for SOC). A second monitor was positioned beside the test screen showing a standard screen saver program. During rest phases, individuals looked at the computer screen, which was running a conventional screen saver (starfield, Microsoft Corp., USA) [12]. Patients and subjects were told to sit in an upright and comfortable position, and to breathe normally. Talking and unnecessary movements were not allowed, and the examiner regularly checked eye movements during tasks and rest, i.e. subjects' eyes had to be either directed towards the presented tasks or the monitor with the resting condition, respectively.

### Cognitive tasks

**Figure 2:**
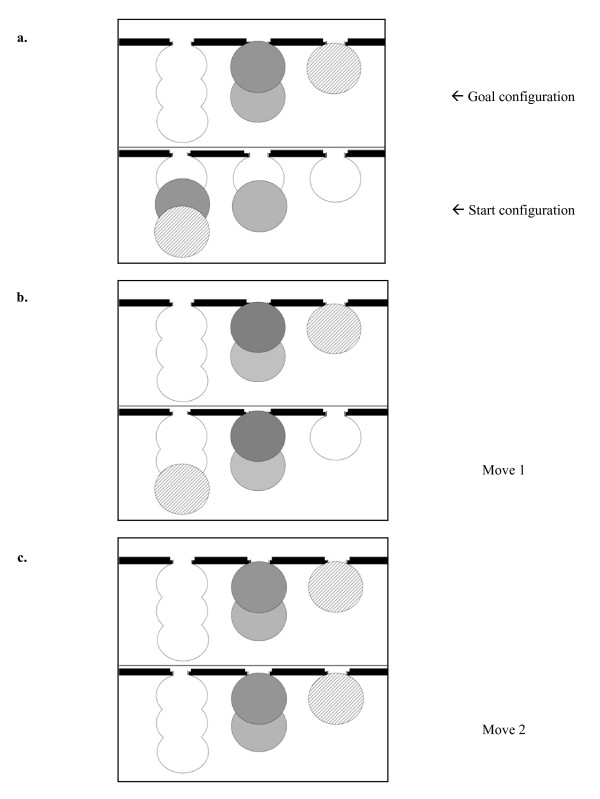
**Stockings of Cambridge**. Schematic illustration of a 2-move problem, with start configuration (**a**.), move 1 (**b**.) and move 2 (**c**.). Note: grey shades and hatching instead of colours.

Stockings of Cambridge: This test has been described previously by our group [11]. Briefly, there was a goal configuration on the upper part of the screen and a start configuration on the lower part, each containing three pockets of different sizes (Fig. 2a). Three differently coloured balls were located in the goal and the start configuration. The subject's task consisted of copying the goal configuration by moving the balls in the start configuration (Fig. 2b, c). The predetermined number of minimum moves differed from task to task to enable the investigation of easy and difficult conditions (2–3 and 4–5 minimum moves, respectively). Subjects moved the balls by selecting them with their right index finger. One block consisted of a 2, 3, 4, and 5-move problem with two different trials per problem, respectively. There was a pause of 20 seconds after every trial. Each block started with a 2-move problem while the following 3, 4 and 5 move problems were presented in random order. Care was taken that problems of a distinct difficulty differed between blocks to avoid learning effects. All subjects had to complete one block per artery pair. If subjects were not able to solve a trial in a determined number of maximum moves (5, 7, 9 and 12 maximum moves for 2, 3, 4 and 5 move problems, respectively), the computer stopped automatically, displaying the next trial after a 20 second pause.

SOC control task: There was an incorporated control task (SOC control task), which required the subject to copy one move at a time in exact correspondence with the moves made in the preceding planning condition. The control task matched the previous planning problems in terms of number of moves and comprised the same number of trials (i.e. 8 planning tasks and 8 control tasks). It was inserted as follows: at the end of the block and only once in between if an easier problem followed a more difficult one (e.g. 2,2 – 5,5 – c2, c2 – c5, c5 – 4,4 – 3,3 – c4, c4 – c3, c3). Hence, the SOC control task was designed to assess the visuomotor component of the SOC task.

WCST: The WCST was presented with four cards at the top and a pile of cards at the bottom, always depicting symbols on the top card [12]. The top card had to be matched with one of the four cards according to one of the rules of colour, shape and number, and feedback was given through a high (i.e. correct) or low (i.e. incorrect) pitched tone. After 6 correct card sorts representing one achieved category, the matching principle was tacitly altered. Each category was presented twice until a maximum of 6 categories or a maximum of 128 trials were achieved. The subject controlled card sorting by means of a conventional computer keyboard, using the fingers of the right hand on the numeric block (keys 1, 2, 3, 4 that indicated the four possible target positions on the computer screen).

WCST control task: Motor and visual activity during WCST was controlled by a control task similar to the one described in [14]: Briefly, subjects were asked to press the same keys (numbers 1–4 of the numeric block on the keyboard), with the same fingers and the same force during 90 s, maintaining constant visual stimulation as described for the resting phases. The frequency of the pressed keys was set at 0.5 Hz (i.e. one beat per 2s, auditory signal) in order to achieve a similar key pressing frequency as during WCST. To assure adequate performance, a practice session was conducted prior to study onset.

### Test sequence

The WCST was repeated once per artery pair, with the WCST control task either before or after the WCST in balanced order. The SOC was applied once per artery pair, and there was also a balanced order between SOC and WCST. Resting periods of 60s duration were inserted between SOC, WCST and WCST control task. Subjects were instructed to stay calm and to relax during resting periods.

### Settings

All subjects received ample instruction about the applied tests and were familiarized with the tasks by solving, in the case of the SOC, several one and two move problems, and in the case of the WCST, one run. These procedures were carried out 30 minutes prior to the start of the fTCD measurements. For the SOC, subjects were instructed to solve the problems efficiently, and to start executing the task after having prepared a plan. For the WCST, patients and subjects were told to sort the cards as swiftly as possible.

### Data collection

Test performance: SOC: The following parameters were measured: 1) adjusted planning time, i.e. the time needed to construct the sequence to solve the task minus the respective initiation time during the SOC control task, providing a "purer" estimate of planning [1], 2) execution of planned movements, i.e. the time spent after having touched the first ball until the completion of the task, 3) the proportion of problems solved using the minimum number of moves provided specific information about task difficulty [8]. Note: Results on performance in healthy subjects suggested that 2 and 3 move problems were mostly solved in the minimum number of moves [11], which was not true for 4 and 5 move problems. Therefore, we differentiated between easy (average of 2 and 3 move problems) and difficult problems (average of 4 and 5 move problems).

WCST: The following performance variables were considered: number of achieved categories, percentage of perseverative errors, reaction time (RT) of 2^nd ^and 3^rd ^trials as means of set shift (note: only RTs were included where, in fact, a set shift had taken place), and RT of 5^th ^and 6^th ^correct trials as means of maintaining set [9].

### MFV

Offline analysis of MFV was carried out as described in [12] and comprised the following steps: a) integration of absolute MFV to one value per heartbeat, b) offline export of the digitized MFV data to a commercially available spreadsheet program (MS-Excel, Microsoft Corp., U.S.), c) normalization of digitized data with reference to pre- and post-task rest conditions [24] (60 s intervals of rest with 30 s between the first and last 15 s, yielding one absolute and averaged baseline MFV; for rest condition see Methods, Technical procedures), d) conversion from heartbeat to second-wise frequency, e) averaging across periods of interest. The normalization procedure correspondingly followed the description of Markus and Boland [24], i.e. the average of resting periods was set at 1, and MFV change during tasks was put in relation to that value, resulting in decimal increase or decrease of MFV or relative MFV per second (note: one might as well describe these alterations as percentage change). These relative MFV values were then averaged for time intervals of interest. All MFV values in this paper are *relative *MFV.

Time intervals of interest: a) SOC: initial 5 s of planning, movement execution and SOC control task; b) WCST and WCST control task (for details see [12]): time interval of 40–70 s after the start. Because only 6 correct trials were necessary to complete one category during WCST, the duration between two set shifts was too short for a return of the MFV to levels during maintaining set and hence to a difference between maintaining set and set shifting. Therefore, this study did not include information on MFV during those phases.

Because of rapid performance of SOC, 10% of the MFV values could not be evaluated during time intervals of 5 s. Missing values were replaced by cells calculated by multiple linear regressions (MLR). This ad-hoc procedure comprised the following steps:

1) Data of patients and healthy subjects were treated separately, because there is evidence that brain perfusion under executive function differs between these groups [14]. Further, missing value analysis (MVA) was carried out separately for hemisphere, first and second trials.

2) All available MFV data of subjects during the initial 5 s (in the (temporal) order of 0 s to 5 s resulting in 6 or less MFV values per subject) of a defined group, hemisphere, number of trial and phase were considered for MLR, and MVA provided by SPSS (Statistical Package for the Social Sciences version 10.0 for Microsoft Windows, SPSS Inc., USA) was performed using maximum likelihood (ML). Data of all subjects entered the model as predictors as well as dependent variables, and regression models were supplemented with a random component (for details, see SPSS documentation, SPSS Inc., USA). The resulting files were then saved, and one mean value per subject was obtained by averaging MFV time intervals of 0–5 s (see above "Offline analysis" of this section).

3) Multiple linear regression with maximum likelihood as MVA method was chosen for several reasons: First, we wanted to employ a method that implemented as much information as possible to develop estimates with desirable properties; second, this method should include a modern method of MVA, such as maximum likelihood. Third, a series of preliminary analyses with this procedure resulted in values that were within a range of the MFV time course to be expected during executive functioning [14].

4) In order to decrease the amount of missing values, smaller MFV time intervals of 4 s and 3 s were chosen (7% and 3% missing values, respectively) and corresponding statistical analyses were carried out. These analyses yielded similar results to those carried out with time intervals of 5 s (data not shown).

5) Missing values of cerebral arteries that could not be insonated because of technical reasons were not replaced.

### Statistical analyses

**Figure 3:**
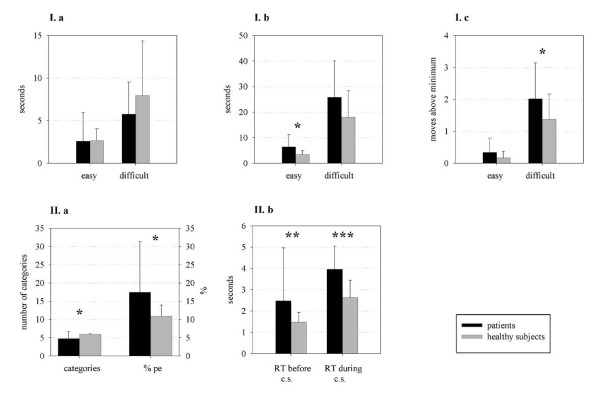
**Performance during the SOC and WCST in patients (n = 22) and healthy subjects (n = 20) **SOC: **I a**. Adjusted planning time during easy and difficult problems, **I b**. Movement execution time, **I c**. Average moves above minimum, WCST: **II a**. Number of categories and percentage of perseverative errors (%pe), **II b**. Reaction time (RT) during maintaining set and set shifting. Abbreviation: c.s.: category shift. *P < 0.05, **P < 0.01, ***P < 0.001.

The Kolmogorov-Smirnov test (KST) was applied to test for normality of distribution (P < 0.05). Perseverative errors and the number of categories were non-normally distributed. Log transformation of perseverative errors resulted in a non-significant result of the KST, and parametric procedures were chosen to examine between-group differences. The number of categories remained non-normally distributed after transformation; therefore, the Mann-Whitney U Test was carried out to examine between-group differences (note: in order to make these variables easier to interpret, untransformed mean values are presented in Fig. 3 II. a). Two-tailed significance testing was used throughout, with an alpha generally set at 0.05. Due to multiple comparisons in correlation analyses, alpha was set at 0.005. Descriptive statistics are presented as mean ± standard deviation.

Between-group differences of performance and MFV: 1) Task performance. For SOC, performance differences between diagnostic groups were assessed by separate repeated measures of univariate analyses of variance (ANOVAs) with diagnostic grouping as a between-subject factor, task difficulty as a within-subject factor (easy and difficult problems) and the respective performance variable (e.g. adjusted planning time) as a dependent measure. For WCST, independent samples t-tests were carried out between diagnostic groups and respective variables as dependent measures. 2) MFV: Since hemisphere was not a significant factor in the following statistical analyses, we averaged MFV values of the left and right hemisphere to one value. SOC: Separate univariate analyses of covariance (ANCOVAs) with diagnostic group as a between-subject factor, MFV (planning, movement execution) of a respective degree of difficulty as a dependent measure and MFV during the equivalent SOC control task as a covariate.

In order to avoid ad-hoc imputations of missing MFV values as described above, we cross-validated results obtained by the General Linear Model (GLM) by means of PROC MIXED (SAS Inc., Cary, NC, USA), which uses maximum likelihood estimation of mixed models and a more general covariance structure approach. For a recent comparison of these two methods, see McCulloch [25]. Since the results of the analyses with PROC MIXED were largely the same, only results from the GLM are presented in the following.

WCST: Separate ANCOVAs with diagnostic grouping as a between-subject factor, MFV during WCST as a dependent measure and MFV during the WCST control task as a covariate. The impact of performance on between-group MFV differences was examined by means of ANCOVAs with diagnostic grouping as a between-subject effect and performance as a covariate.

There is evidence that higher cognitive functions provoke distinct MFV patterns in the left and right MCA of men and women [26]. In order to examine this important aspect of brain perfusion and in an attempt to keep the threshold of significance low due to the small number of individuals, simple independent sample t-tests were applied to examine significant MFV differences with gender as a between-subject effect, separately for the artery side and diagnostic group.

Associations of performance: Zero-order correlation analyses using Pearson's product moment correlation coefficient were applied to assess the linkage between SOC and WCST. a) SOC variables: adjusted planning time, movement execution time, b) WCST variables: RT before and during set shifting. Fisher's r to z transformations were used to examine between-group differences of r [27].

Associations of MFV: Pearson's product moment correlation coefficient was used to assess the linkage between MFV in SOC and WCST. a) SOC variables: MFV during planning and movement execution. b) WCST variables: MFV during 40–70 s. Significant correlations were controlled for: a) SOC control task and b) respective time intervals in WCST control task. Between-group differences of r were investigated with Fisher's r to z transformations [27]. Moderator variables: In patients with schizophrenia, significant associations were controlled for a set of clinically relevant covariates, using a priori knowledge from studies that reported significant effects on executive function [1,6]. Applying separate partial correlation analyses, the following covariates were deemed as relevant: premorbid IQ, duration of illness, total BPRS score, dosage of antipsychotics, EPS- and BAS-scores.

## Results

MFV was measured in 22 pairs of MCA, 18 pairs of ACA and one left ACA in patients with schizophrenia. In healthy subjects, 20 pairs of MCA, 17 pairs of ACA and one left ACA were insonated. Due to an error in data collection, no MFV results were obtained in one patient. As subjects whose ACAs gave no sufficient signal did not have any clinical complaints, we included MCA measurements of those individuals in the analysis.

### Performance

Performance data is presented in Fig. 3.

SOC: Adjusted planning time with a significant effect of problem difficulty (F(1,40) = 34.71, P < 0.001, ES = 0.47). Movement execution with a significant between-group effect (F(1,40) = 5.23, P = 0.03, ES = 0.12), and a significant effect of problem difficulty (F(1,40) = 88.88, P < 0.001, ES = 0.69). Average moves above minimum with a significant effect of diagnostic grouping (F(1,40) = 5.51, P = 0.02, ES = 0.12), and a significant effect of problem difficulty (F(1,40) = 92.28, P < 0.001, ES = 0.70). Patients showed normal planning times and somewhat impaired accuracy (average moves above minimum) and movement execution times. Accuracy was decreased during difficult problems, and movement execution times were longer during easy problems, by trend also for more difficult problems.

WCST: Patients were slower during maintaining set and set shifting, they demonstrated more perseverative errors and achieved a somewhat lower number of categories (Fig. 3).

### Association of performance

Results of correlation analyses are presented in Tables 2a, b: Patients showed a significant and positive correlation between movement execution of easy problems and RT during maintaining set. In healthy subjects, we found positive and significant associations predominantly between difficult problems of planning and movement execution and set shifting, as well as a highly significant and positive correlation between movement execution of difficult problems and maintaining set. Fisher's r to z transformations yielded the following significant between-group differences in correlation coefficients: Adjusted planning time during easy problems and RT during set shifting (z = 2.48, P = 0.01) and movement execution time and RT during maintaining set (z = 2.27, P = 0.02). The linkage between both forms of executive tasks was stronger in healthy subjects as compared to patients with schizophrenia.

**Table 2:** Associations between performance parameters of SOC and WCST

**a) Healthy subjects (n = 20)**			
SOC	Task difficulty	RT before shift	RT after shift

Adjusted planning time	Easy	0.37	0.63*
	Difficult	0.39	0.62*
Movement execution time	Easy	-0.05	0.19
	Difficult	0.75**	0.65*

**b) Patients (n = 22)**			

Adjusted planning time	Easy	0.28	-0.10
	Difficult	0.41	0.35
Movement execution time	Easy	0.61*	0.19
	Difficult	0.35	0.40

*P < 0.005, **P < 0.001

Values represent Pearson's product number correlation coefficient

Abbreviations: SOC: Stockings of Cambridge; WCST: Wisconsin Card Sorting test

### MFV

Results are presented in Table 3. We observed decreased MFV in the MCA during planning of easy and difficult problems in patients, and the larger difference during easy problems was due to the fact that healthy individuals showed increased MFV as compared to difficult problems [11], whereas patients' MFV remained the same during planning of easy and difficult problems. Performance variables (movement execution, movements above minimum) were not significant covariates of MFV differences (P > 0.05). For the sake of the focus and readability of this article, systematic analyses of measures of cerebral hemodynamics (such as maximum flow velocities and parameters of time) will be published elsewhere. Former reports by this group have presented information on cerebral hemodynamics in healthy subjects and patients with schizophrenia during different measures of executive functioning [11,12,14].

**Table 3:** Relative MFV during SOC and WCST in patients and healthy subjects.

SOC	artery	patients	95% CI	healthy subjects	95% CI	df	F	ES
*Planning*								
easy	MCA	1.0235 (0.0387)	1.0117 – 1.0416	1.0691 (0.0567)	1.0505 – 1.0811	2,40	13.71***	0.27
difficult		1.0138 (0.0354)	1.0043 – 1.0335	1.0486 (0.0573)	1.0283 – 1.0583	2,40	5.50*	0.12
								
*Mov. exe*.								
easy	MCA	1.0767 (0.0458)	1.0600 – 1.0991	1.0891 (0.0616)	1.0661 – 1.1062	2,40	n.s.	-
difficult		1.0562 (0.0526)	1.0356 – 1.0843	1.0742 (0.0667)	1.0453 – 1.0951	2,40	n.s.	-
								
*Planning*								
easy	ACA	1.0184 (0.0346)	1.0009 – 1.0399	1.0490 (0.0532)	1.0213 – 1.0592	2,34	n.s.	-
difficult		1.0180 (0.0474)	0.9961 – 1.0406	1.0339 (0.0804)	1.0013 – 1.0446	2,34	n.s.	-
								
*Mov. exe*.								
easy	ACA	1.0506 (0.0509)	1.0330 – 1.0731	1.0597 (0.0518)	1.0380 – 1.0769	2,34	n.s.	-
difficult		1.0564 (0.0651)	1.0286 – 1.0847	1.0652 (0.0737)	1.0377 – 1.0922	2,34	n.s.	-

WCST	artery	patients	95% CI	healthy subjects	95% CI	df	F	ES

steady-state	MCA	1.0610 (0.0479)	1.0414 – 1.0805	1.0751 (0.0493)	1.0546 – 1.0948	2,40	n.s.	-
steady-state	ACA	1.0363 (0.0492)	1.0210 – 1.0641	1.0494 (0.0550)	1.0215 – 1.0647	2,34	n.s.	-

values are mean (SD)

*P = 0.024, ***P = 0.0007, all other p-values >0.14

Abbreviations: CI: confidence interval; ES: effect size (ES shown only for significant results); MFV: mean flow velocity; Mov. exe.: movement execution; n.s.: not significant; SOC: Stockings of Cambridge; WCST: Wisconsin Card Sorting Test.

We could not detect consistent gender differences (Tables 4 and 5), although a careful inspection of the results in Table 5 revealed that female patients had subtly higher MFV in the right MCA than male patients, especially during difficult problems of SOC and during WCST (note: the significant MFV increase in the right MCA during WCST in female patients did not remain valid when observing a more stringent significance criterion (alpha set at 0.01) using a Bonferroni correction for multiple comparisons).

**Table 4:** Relative MFV in the MCA during SOC and WCST in healthy subjects, separately presented for hemisphere/diagnostic grouping and gender

SOC	side	men	women	mean diff.	95% CI	df	t-value
*Planning*							
easy	Left	1.0708 (0.0588)	1.0595 (0.0600)	0.0113	-0.0449 – 0.0674	18	0.42
difficult	MCA	1.0617 (0.0560)	1.0271 (0.0646)	0.0347	-0.0228 – 0.0922	18	1.27
							
*Planning*							
easy	Right	1.0702 (0.0627)	1.0765 (0.0586)	-0.0063	-0.0634 – 0.0508	18	0.23
difficult	MCA	1.0542 (0.0651)	1.0548 (0.0587)	-0.0007	-0.0588 – 0.0575	18	0.02
							
*Mov. exe*.							
easy	Left	1.0866 (0.0437)	1.0867 (0.0719)	-0.0001	-0.0577 – 0.0575	18	0.00
difficult	MCA	1.0843 (0.0588)	1.0622 (0.0841)	0.0220	-0.0478 – 0.0919	18	0.66
							
*Mov. exe*.							
easy	Right	1.0842 (0.0522)	1.0977 (0.0812)	-0.0135	-0.0794 – 0.0524	18	0.13
difficult	MCA	1.0791 (0.0540)	1.0702 (0.0780)	-0.0041	-0.0689 – 0.0607	18	0.14

WCST	side	men	women	mean diff.	95% CI	df	t-value

steady-state	Left MCA	1.0887 (0.0707)	1.0750 (0.0571)	0.0136	-0.0463 – 0.0736	18	0.48
steady-state	Right MCA	1.0730 (0.0344)	1.0657 (0.0563)	0.0074	-0.0378 – 0.0525	18	0.34

values are mean (SD)

All p-values >0.2

Abbreviations: CI: confidence interval; MFV: mean flow velocity; Mov. exe.: movement execution; n.s.: not significant; SOC: Stockings of Cambridge; WCST: Wisconsin Card Sorting Test

**Table 5:** Relative MFV in the MCA during SOC and WCST in patients, separately presented for hemisphere/diagnostic grouping and gender

SOC	side	men	women	mean diff.	95% CI	df	t-value
*Planning*							
easy	Left	1.0198 (0.0402)	1.0283 (0.0456)	-0.0085	-0.0492 – 0.0322	19	0.44
difficult	MCA	1.0128 (0.0367)	1.0139 (0.0435)	-0.0011	-0.0389 – 0.0366	19	0.06
							
*Planning*							
easy	Right	1.0158 (0.0396)	1.0415 (0.0426)	-0.0257	-0.0650 – 0.0136	19	1.37
difficult	MCA	1.0104 (0.0337)	1.0226 (0.0506)	-0.0122	-0.0508 – 0.0264	19	0.66
							
*Mov. exe*.							
easy	Left	1.0747 (0.0544)	1.0855 (0.0470)	-0.0108	-0.0614 – 0.0397	19	0.45
difficult	MCA	1.0438 (0.0482)	1.0850 (0.0435)	-0.0413	-0.0866 – 0.0040	19	1.91
							
*Mov. exe*.							
easy	Right	1.0692 (0.0524)	1.0870 (0.0339)	-0.0177	-0.0636 – 0.0282	19	0.81
difficult	MCA	1.0398 (0.0623)	1.0847 (0.0474)	-0.0449	-0.1012 – 0.0113	19	1.67

WCST	side	men	women	mean diff.	95% CI	df	t-value

steady-state	Left MCA	1.0559 (0.0381)	1.0868 (0.0572)	-0.0308	-0.0745 – 0.0128	19	1.48
steady-state	Right MCA	1.0388 (0.0489)	1.0889 (0.0495)	-0.0501	-0.0976 – -0.0026	19	2.20*

values are mean (SD)

*P = 0.04, all other p-values >0.07

Abbreviations: CI: confidence interval; MFV: mean flow velocity; Mov. exe.: movement execution; n.s.: not significant; SOC: Stockings of Cambridge; WCST: Wisconsin Card Sorting Test

### Association of MFV

**Figure 4:**
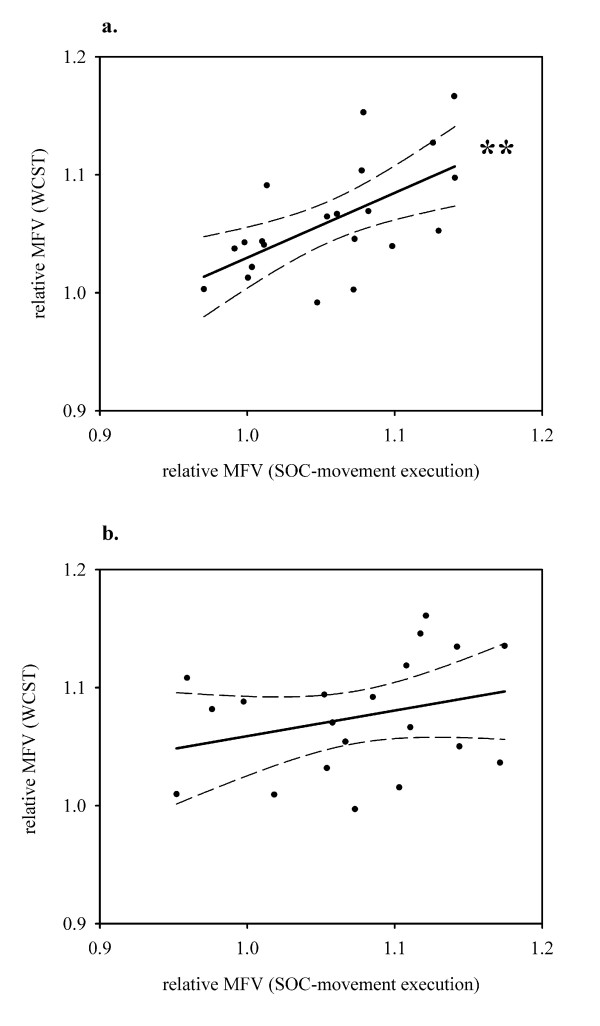
**Association of MFV in the MCA during movement execution of SOC and steady state of WCST. a**. Patients (n = 21), Beta = 0.60, **P = 0.004. **b**. Healthy subjects (n = 20), Beta = 0.29, P = 0.21. Solid line: linear regression, dashed lines: 95% confidence intervals.

Significant correlations in the MCA were restricted to patients with schizophrenia: there was a positive association between MFV during movement execution of difficult problems and MFV during WCST (R = 0.60, P = 0.004) (Fig. 4), and it remained significant by trend when controlling for the SOC control task (P = 0.008), but attenuated slightly when controlling for the WCST control task (P = 0.02). However, the WCST control task should be considered as a relatively unspecific sensorimotor stimulus. Moderator variables did not alter this significant correlation. There was no significant z-value between diagnostic groups (P > 0.1).

## Discussion

The main results of this study on the association between two tasks of executive function in schizophrenia can be summarized as follows: First, patients showed an aberrant association pattern between SOC and WCST performance, and there was only one significant correlation: longer movement execution time during easy problems of SOC was associated with slowing during maintaining set of WCST. Second, there was a significant association between MFV in SOC and WCST, confined to the MCA in schizophrenia. Third, patients showed decreased MFV in the MCA during planning of SOC.

### Performance

In patients with schizophrenia, the observed deficits in performance during SOC are in agreement with results presented by Morris et al. [1]: unimpaired planning times, deficits in accuracy and movement execution times, and a strong effect of task difficulty. According to Morris et al. [1], performance deficits during planning are similar to those found in patients with frontal lobe lesions [8]. However, the pathophysiological substrate of schizophrenia is not restricted to the frontal lobes, but likely involves multiple brain areas (for review, see [28]). Concerning the effect of task difficulty during SOC, the disproportionate increase of movement execution times during difficult problems in both groups may be due to the fact that subjects made an incomplete plan, and were forced to re-plan during movement execution [11]. WCST performance was decreased in patients with schizophrenia, which is in agreement with results reported by others [2,29].

There was only one significant and positive association between SOC and WCST in schizophrenia (movement execution time of *easy *problems and RT during maintaining set), which was not found in healthy subjects. In the latter group, more numerous and distinct correlations were detected, particularly, three out of four significant associations were between SOC and set shifting of WCST. Set shifting has been implicated with the need to change tasks. Monsell [30] stated that subjects' responses immediately after task switch were slower and more error-prone and he suggested that "switch cost" results from both transient and long-term carry-over of "task-set" activation and inhibition and time consumed by task set reconfiguration. On the other hand, disproportionally prolonged movement execution times for difficult problems (approximately up to 5 times longer than for easy problems, see Fig. 3, Ib) may be due to the fact that subjects were forced to create a new plan because the planned sequence was incomplete – so called online planning [31,32]. More rapid planning and carrying out a plan showed shared variances of up to 42% with higher speed during set shifting, implying that set shifting requires mental planning and also online planning such as observed in difficult problems of SOC. More generally, one could argue that set shifting of WCST resembles a difficult planning paradigm including components of mental and online planning. Shorter movement execution for difficult problems was also linked to higher speed during maintaining set, suggesting that maintaining set implements features of online planning with a shared variance of 57%, or, somewhat surprisingly, that maintaining set is a demanding process. The findings of our study advocate the notion that maintaining set and set shifting represent two distinct and rather difficult phases of WCST. In other words, the WCST is a difficult task of executive functioning. The significant associations between SOC and WCST in healthy subjects are not primarily intuitive, since both tasks have been brought into context with distinct neuropsychological constructs. Spatial abilities and problem solving are thought to be a central feature of SOC [4], whereas WCST includes abstraction, rule maintenance and rule learning [3]. The SOC has a clear und unambiguous goal state, in contrast to the trial-wise feedback mode with rule shifting properties of the WCST. Attention and working memory play a pivotal role during WCST [33,34] and during SOC [35], and, as our study demonstrates, a joint effort of mental and online planning are, among other components, necessary for set shifting. In schizophrenia, deficits of working memory and attention have been well documented [36], and we propose that these patients are unable to properly elaborate a joint effort to solve difficult executive tasks, illustrated by smaller shared variances of SOC and WCST performance. A hint in a similar direction might be the aberrant association between movement execution during *easy *problems and maintaining set in patients, a linkage that was significantly different from healthy subjects. This finding suggests that patients use inappropriate strategies aimed at solutions for difficult problems during maintaining set, providing a further explanation for lower performance during WCST in schizophrenia. Overall, the results of performance associations during executive tasks in schizophrenia imply, in the case of difficult SOC problems, a lack of associations with WCST, and in the case of easy SOC problems, dysfunctional relations with WCST. This may be due to the fact that patients have either a lower capacity to solve more complex problems, or because there is a dissociation of executive functions in this disorder [2].

### MFV

In patients with schizophrenia, mental planning provoked a MFV decrease in the MCA that was not found in the ACA. This result suggests a dysfunctional brain perfusion during mental planning within the territory of the MCA, i.e. the lateral hemispheres. Brain imaging studies have shown that activation of the dorsolateral prefrontal cortex (DLPFC) is a central feature during planning in normals [37,38]. However, neuroimaging evidence suggests the involvement of other regions during planning [37,38] and other executive tasks (Stroop-like tasks, transitive inference and concept formation) [39-41], such as areas within the prefrontal, parietal and temporal cortex, including also the cingulate cortex and subcortical areas. There is evidence that prefrontal areas and particularly the DLPFC are dysfunctional during executive function in schizophrenia [5]. Based on the findings of our study and those reported in available neuroimaging literature on planning [37,38], we suggest that lower MFV in the lateral hemispheres is at least in part due to lower flow within the DLPFC. This result cannot directly be compared to observations of other corresponding studies [6,42], where also a prefrontal involvement has been observed in schizophrenia, because even a very recent report on TOL in schizophrenia used a forced choice paradigm that did not differentiate between planning and movement execution [42]. Further studies are required to elaborate the complex concept of so-called "hypofrontality" in schizophrenia, referring to the lower brain perfusion in prefrontal regions during executive tasks described in earlier neuroimaging studies [5,6]. A recent review suggested that hypo- or hyperfrontality in schizophrenia is a rather dynamic feature [43], depending on task load, and Ragland et al. [44] proposed that patients with schizophrenia have abilities to engage lateral prefrontal areas when organizational demands are reduced. Further, a meta-analysis on n-back tasks in this disorder found evidence of lateral hypofrontality and medial hyperfrontality [45]. While our report did not resolve this important issue, fTCD should be regarded as a potentially useful tool since MFV in lateral and medial parts of the brain can be measured separately. This study detected, to the best of our knowledge for the first time, reduced brain perfusion in lateral parts of the brain during mental planning of SOC in chronic schizophrenia.

Statistical analyses revealed only one significant and positive MFV association in the MCA of patients with schizophrenia, namely between execution of *difficult *problems and the steady state of the WCST. Steady state of WCST means that maintaining set and set shifting were averaged (see Methods, MFV), and this linkage cannot differentiate between those distinct processes. Two aspects seem of relevance when looking at this association: First, there were no MFV differences between diagnostic groups during carrying out difficult SOC problems and WCST (Table 3); second, on the behavioural level, patients did not show any significant associations between difficult SOC problems and WCST, markedly contrasting findings in healthy subjects (Table 2). At this point, it can only be speculated that carrying out difficult SOC problems and WCST activate the same areas in the lateral brain in schizophrenia, and based on the above discussion, probably the DLPFC *without *lower brain perfusion. These findings corroborate the notion that the relationship between performance of "prefrontal" tasks and MFV might be more complex, there not being a simple linear relationship such as hypofrontality resulting in low performance in schizophrenia. We suggest that neural networks involved in planning and execution of a plan differ between normals and schizophrenia. Furthermore, the restricted spatial resolution of fTCD should be taken into account when interpreting the results of our study. Clearly, neuroimaging studies with a high spatial and temporal resolution could potentially resolve issues on altered circuits during separate phases of a planning and a rule generation task in schizophrenia. To the best of our knowledge, no such investigations have been carried out to date.

This study has the following limitations: First, the insufficient temporal bone window prevented the insonation of the ACA in a few patients and subjects, which is a well-known drawback of TCD. Second, our patients were treated with antipsychotics, and we cannot rule out that our results were not influenced by this medication. We consider this limitation as relatively minor, since most patients were on atypical antipsychotics that have only a minor impact on striatal dopamine D2 metabolism [46], and moreover there is evidence that antipsychotic medication normalizes vasoreactivity in schizophrenia [47]. Third, we did not control for general motor slowing of patients during WCST; however; we can rule out motor slowing as a cause of significant associations comprising planning in the sense of adjusted planning time. Fourth, the comparison of correlations between diagnostic groups yielded significant differences in only a relatively low number of cases, despite conservative thresholds of alpha levels. Further, future studies should implement larger samples for Fisher's r to z transformations.

## Conclusion

This study revealed an aberrant pattern of behavioural and lateral brain perfusion associations in schizophrenia during two well-known but different tasks of executive functioning. Further, we found lower brain perfusion in lateral hemispheres during mental planning in schizophrenia. This study supports the notion that executive dysfunction in schizophrenia involves lateral parts of the brain. These unprecedented results also emphasize the scientific potential of fTCD in assessing brain perfusion during executive functions in psychiatric populations.

## Competing interests

The author(s) declare that they have no competing interests.

## Authors' contributions

DF helped carrying out the experiment, was involved in major parts of data handling, and drafting of the manuscript. DS carried out the experiment, supervised data handling and manuscript drafting. BvR was involved in data transformation and data preparation, and she helped drafting the manuscript. AT helped on methodical issues of this study and helped drafting the manuscript. DH participated in the design and coordination of the study, interpretation of the data and drafting of the manuscript.

## Pre-publication history

The pre-publication history for this paper can be accessed here:


